# Policy Series: 3 Pilot Collaborations to Empower Nursing Home Residents and Staff: Taking Action With the Moving Forward Coalition

**DOI:** 10.1093/geroni/igaf122.1982

**Published:** 2025-12-31

**Authors:** Mairead Painter, Anna Fisher

## Abstract

The Moving Forward Nursing Home Quality Coalition brings together a diverse group of nursing home stakeholders to advance recommendations from “The National Imperative to Improve Nursing Home Quality,” a landmark 2022 report by the National Academies of Sciences, Engineering, and Medicine (NASEM). A core part of the Coalition’s mission is designing, testing, and spreading innovative and accessible solutions to support and empower nursing home residents and direct-care staff. This symposium presents three such initiatives that translate national recommendations into actionable strategies with implications for quality improvement and long-term care policy. A partnership with the Kansas State University Center on Aging supports up to 30 nursing homes in implementing tools to assess and act on residents’ goals of care, promoting alignment between care delivery and individual priorities. A second initiative, led in collaboration with the Connecticut Long-Term Care Ombudsman Program, strengthens the role of resident councils by promoting statewide adoption, disseminating best practices, and integrating resident feedback into nursing home quality improvement processes. The third initiative convenes a national learning network of Geriatrics Workforce Enhancement Program (GWEP) grantees to develop shared standards for certified nursing assistant (CNA) apprenticeship programs, with the aim of strengthening the direct care workforce and improving job quality. Presenters will share insights from their first year of implementation, including consensus-building strategies, early outcomes, and lessons learned. Together, these efforts reflect the Coalition’s broader commitment to making change and system-level improvement through a collaborative process.

